# The Overall Quality Changes of Chinese Sauced Ducks at Different Stages During Processing and Storage

**DOI:** 10.3390/foods14050834

**Published:** 2025-02-28

**Authors:** Kaiyong Yao, Jie Cai, Daodong Pan, Bindan Chen, Jinghui Fan, Daxi Ren, Yingping Xiao

**Affiliations:** 1 State Key Laboratory for Quality and Safety of Agro-Products, Institute of Agro-Product Safety and Nutrition, Zhejiang Academy of Agricultural Sciences, Hangzhou 310021, China; yao1234@zju.edu.cn; 2 Institute of Dairy Science, College of Animal Science, Zhejiang University, Hangzhou 310058, China; zjcaijie@zju.edu.cn; 3 Lanhai Ecological Agriculture Co., Ltd., Hangzhou 311402, China; 4 Department of Veterinary Medicine, College of Animal Sciences, Zhejiang University, Hangzhou 310029, China; 5 College of Food Science and Engineering, Ningbo University, Ningbo 315211, China; daodongpan@163.com; 6 Zhejiang Guowei Technology Co., Ltd., Zhuji 311800, China; chenbindan@163.com; 7 Institute of Animal Sciences, Hangzhou Academy of Agricultural Science, Hangzhou 310024, China; ufanta@163.com

**Keywords:** sauced duck, flavor change, microbial change, physical-chemical change, sensory change, flavor development

## Abstract

This study reveals the physicochemical, microbial, flavor, and sensory changes in sauced duck from the marinating phase to the end of storage, divided into six stages (stages A–F). The changes in color, total plate count, total volatile basic nitrogen, and thiobarbituric acid reactive substance at different stages were clarified. We utilized 16S rRNA gene sequencing, GC-IMS, and GC-MS to explore the changes in bacterial flora, fatty acid composition, and flavor characteristics. The dominant bacteria identified in stages A–C included *Psychrobacter*, *Flavobacterium*, and *Pseudomonas*, while *Lactobacillus* and *Staphylococcus* dominated during stages D–F. Aldehydes, esters, alcohols, and ketones emerged as the main flavor compounds. Several unsaturated fatty acids significantly (*p* < 0.05) decreased from stage A to stage F. The sensory quality of sauced duck improved. The potential reactions were determined, and correlation analysis of sauced duck samples across different stages was performed. 3-Methy-1-butanol could be a crucial indicator of sauced duck’s overall quality. This research could support the treatment optimization of sauced duck products.

## 1. Introduction

China has the highest consumption of duck meat globally, accounting for over 70% of global production [1,2]. In Zhejiang Province, locals typically select Shaoxing pockmark duck (*Anas platyrhyncha var. domestica*) as raw material for sauced duck. Chinese consumers widely favor this traditional sauced duck product for its delicious taste, unique flavor, and desirable textural properties. Duck meat is rich in high-quality protein and poly-unsaturated fatty acids (PUFA), including C18: 2 and C18: 3 [3]. The processing methods of sauced duck vary across different regions in China. Therefore, sauced duck’s flavor compounds and sensory qualities may differ due to different processing methods [3,4]. In Zhejiang Province, the cooking and drying treatments for sauced duck are performed after marinating, which brings an intense aroma and delicious taste and is highly favored by consumers [2]. Additionally, the natural air drying and sun drying during storage could strengthen the aroma and taste of sauced duck.

However, previous studies of duck meat have yet to deal with the flavor and sensory development during processing and storage. The processing properties of Shaoxing duck need to be further explored. Sauced duck is highly vulnerable to bacterial growth and spoilage due to its high moisture content [5]. The flavor development might be highly related to bacterial flora, especially during storage. The mechanism behind the changes in sauced duck during processing and storage must be elucidated, especially from the perspective of the phase from marinating to storage.

Therefore, this work aimed to investigate the physicochemical, microbial, chemical, and flavor changes in sauced ducks from the marinating treatment to the end of storage. The change in flavor compounds was clarified, and relevant potential correlations were further analyzed. This work could provide a basis for optimizing the processing and storage of sauced duck and improving its overall quality.

## 2. Materials and Methods

### 2.1. Chemicals and Reagents

The aqueous solutions were prepared using ultrapure water. All chromatographic-grade reagents were purchased from Sigma-Aldrich Chemical Co., Ltd. (St. Louis, MO, USA). Other analytical grade chemicals and reagents were all purchased from Aladdin Co., Ltd. (Shanghai, China). All spice food additives were food-grade.

### 2.2. Sauced Duck Preparation

Lanhai Ecological Agriculture Co., Ltd. (Hangzhou, Zhejiang Province, China) provided fresh duck carcasses and the main production methods of sauced duck. According to the local policies in Hangzhou City, three-year-old laying Shaoxing pockmark ducks were slaughtered in the official slaughterhouse. After an approximate time interval of 6–12 h, the following production processes were carried out: marinating, cooking, drying, and preserving. The marinating ingredients contained more than ten spices (cinnamon, star anise, allspice, pepper, etc.). The carcasses were marinated in the mixture for 72 h and were flipped from one side to the other every 24 h. The packet was boiled with mixed seasoning (including 15% soy sauce, 9% yellow wine, and 3% white sugar) for 10 min during the cooking process. Yellow wine is a national wine and seasoning in China and is made from rice, millet, and other grains [6]. The drying treatment was conducted within a 50–60 °C temperature range for 15 h. All dried samples were stored at room temperature (25 ± 1 °C) and humidity (55 ± 5%). Samples were exposed to stable air circulation and natural light on sunny days (10:00–16:00, 6 h), based on the chief engineer’s experience. The chief engineer is the supervisor of the processing workers. Standardized procedures and dedicated supervision ensured the sauced duck samples’ uniformity and quality.

### 2.3. Experimental Group Design

Twelve parallel samples were collected from 72 Shaoxing pockmark duck samples at six stages. All main body parts (including duck leg, breast, and wing) were tested for all indexes, and the mean values were calculated. The processing and storage treatment of all parallel samples was the same. A–F represents each of the six sampling stages in sequential order. Concretely, stage A stands for after marinating and before cooking; B stands for after cooking and before drying; C stands for samples collected after drying and before storage; D, E, and F stand for samples stored for 5, 10, and 15 d, respectively.

### 2.4. Determination of Edible Indexes

The color, total plate count (TPC), total volatile basic nitrogen (T-VBN), and thiobarbituric acid reactive substance (TBARS) values are essential indexes of evaluating whether the sauced duck is edible.

Color: We measured the surface color values of the duck sample with a Minolta colorimeter (CR-10 Plus, Minolta, Japan).

TPC: TPC was strictly determined according to our previous study [5]. Briefly, 25 g of sample was placed into 225 mL of sterile 0.85% sodium chloride (NaCl) solution and homogenized in a bag mixer (Inter-science Ltd., Cantal, France) for 2 min. After 10-fold serial dilution, 1 mL of the sample was plated on agar plates (Land Bridge Co., Ltd., Beijing, China) and incubated at 37 °C for 48 h. The TPC is expressed as log_10_ CFU/g meat.

T-VBN: T-VBN was determined according to Liu et al. [5]. Briefly, 20 g of sample was homogenized in 2% trichloroacetic acid at 5000 rpm for 1 min. The mixture was filtered and added to 1% magnesium oxide (MgO) solution. The liberated T-VBN was absorbed utilizing a 2% boric acid solution and titrated with a 0.01 N hydrochloric acid (HCl) solution. The result is expressed as mg/100 g of sample.

TBARS: We analyzed the value of TBARS with malondialdehyde (MDA) assay kit (Jiancheng Ltd., Nanjing, China) following the manufacturer’s instruction, and the result is expressed as mg of MDA per kg.

### 2.5. Fatty Acid Analysis

The fatty acid (FA) composition was determined according to the method of [7]. The minced sample was added to chloroform–methanol solution (1:2:0.8, *v*/*v*/*v*) and extracted for the fat content using saturated salt water. After saponifying fat using a sodium hydroxide (NaOH)–methanol (CH_3_OH) mixture under reflux in a water bath (85 °C), we removed the water using sodium sulfate (Na_2_SO_4_) and dissolved it with hexane. A 7890B-7000C gas chromatography–mass spectrometry (GC–MS) system (Agilent, Santa Clara, CA, USA) equipped with a capillary column (CD-2560, 100 m × 250 μm × 0.20 μm; CNW, Dusseldorf, Germany) was utilized. The FA were identified using the NIST 14 database. In this study, FAs was classified as saturated fatty acid (SFA), mono-unsaturated fatty acid (MUFA), and PUFA.

### 2.6. Sensory Evaluation

The method from [8] was followed. The sensory evaluation was conducted by ten professionally trained panelists aged 20–30, including five males and five females. We signed an agreement with all participants to use and study their information. In line with the agreement, all participants’ privacy rights are fully protected. All duck meat samples were water-rinsed, cut into even-sized pieces, and placed into clean glass jars. In order to simulate the general consumption mode of sauced duck, these samples were steamed in boiling water until the core temperature exceeded 75 °C. All cooked samples were provided as blind samples. Panelists could not communicate with each other during the sensory evaluation. The concrete criteria are shown in Table S1. Before each tasting, all panelists rinsed their mouths thoroughly to ensure no residues interfered. The study includes only anonymous data. All tested samples in this study were safe for human health. Therefore, this study did not require ethical review board approval according to the regulations of Chinese government.

### 2.7. Microbial Community Analysis

The 16S rRNA gene sequencing was performed based on the previous studies [9]. Sequencing libraries were generated using the Next^®^ Ultra™ DNA Library Prep Kit for Illumina (New England Biolabs, Ipswich, MA, USA). The library quality was assessed using the Qubit 2.0 Fluorometer (Thermo Fisher Co., Waltham, MA, USA) and Bioanalyzer 2100 system (Agilent Co., Santa Clara, CA, USA). The library was sequenced on the Mi-Seq platform (Illumina Co., San Diego, CA, USA). Paired-end reads from the original DNA fragments were merged using FLASH (Johns Hopkins University, Baltimore, MD, USA). Sequences analysis was performed by UPARSE (Independent Investigator, CA, USA), according to [10].

Sequences with more than 97% similarity were assigned to the same OTUs. The relative abundances were visualized using the Krona chart referred to by [11]. The heat map was drawn with software R 4.2.2. Effect size (LEfSe) analysis was conducted by Python LEfSe package (Python 3.6.6). ANOSIM and MRPP were performed based on the Bray–Curtis dissimilarity distance matrices. Species with an LDA score of more than four were identified as biomarkers. Rarefaction curves were found based on these metrics. We calculated three metrics: Chao1, Simpson, and the Shannon index. Cluster analysis was preceded by principal component analysis (PCA) using the QIIME (version 1.8.0, Knight Lab, University of California, San Diego, CA, USA). We used unweighted unifrac distance for PCoA and UPGMA clustering.

### 2.8. Gas Chromatography–Ion Mobility Spectrometry Analysis

The volatile flavor compounds were determined by a Flavor spec^®^ gas chromatography–ion mobility spectrometry (GC-IMS) system (G.A.S, Dortmund, Germany) equipped with an MXT-WAX column (30 m × 0.53 mm × 0.1 μm, Restek, PA, USA). Samples were incubated at 60 °C for 20 min, and then, 500 µL of gas was injected into the injector. After being ionized, the analyte was added to the ionization chamber. The main parameters were 500 V/cm of linear voltage, 45 °C of drift tube temperature, and 75 mL/min of drift gas flow rate. Nitrogen gas (purity > 99.999%) was used as the carrier gas. The programmed flow was set as follows: 2 mL/min maintained for 2 min, then increased to 10 mL/min within 8 min, raised to 100 mL/min in the next 10 min, and then 100 mL/min maintained for 20 min.

We analyzed measurement data in IMS using the VOCal software 0.4.03. The retention index was calculated according to the retention time and ion migration time of volatile substances in GC. The substances were clarified by matching the GC-IMS database and NIST 2020. The description of flavor compounds was identified according to the Flavor Ingredient Library (https://www.femaflavor.org/flavor-library, accessed on 3 November 2024).

### 2.9. Statistical Analysis

The present study employed ANOVA analysis of variance and an independent *t*-test to assess differences in the groups, utilizing Duncan’s test from SPSS Statistics 26.0 (IBM Co., New York, NY, USA). The significance level for this study was established at *p* < 0.05. The Pearson correlation analysis, PCA, and partial least squares–discriminant analysis (PLS-DA) were calculated by SAS 9.1.3 Software (SAS Institute Inc., Cary, NC, USA). The heat maps were generated by Graphpad Prism Software 8.3.0.

## 3. Results and Discussion

### 3.1. Chemical and Physical Changes of Samples Across Different Stages Were Analyzed

The color, TPC, T-VBN, and TBARS results are shown in Table 1. Color is a crucial sensory indicator for sauced duck and affects the human appetite. In this study, the marinating and drying treatment significantly (*p* < 0.05) influenced the L* values. The minor changes of a* values might be due to the marinating treatment. Duck meat products are ideal mediums for microbial growth [5,12]. The unpacked samples with high water activity were vulnerable to microbial growth and contamination, which accounted for the significant increase (*p* < 0.05) in TPC during stages A–F. Cooking and drying treatment could inhibit microbial growth by regulating temperature and humidity. The TVBN and TBARS significantly (*p* < 0.05) increased during stages D–F (Table 1). T-VBN, which is highly correlated with an off-odor, is a critical indicator of spoilage for meat samples [13,14]. The high T-VBN and TBARS values at stage F deserved extra attention.

**Figure 1:**
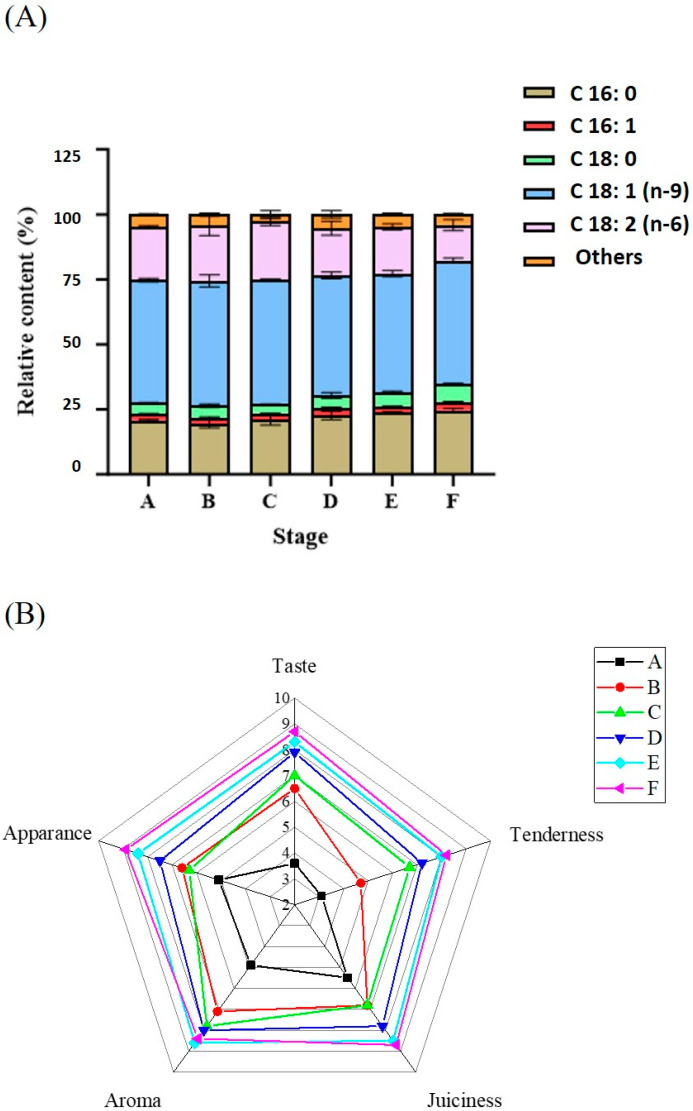
Free fatty acids analysis (**A**) and sensory evaluation (**B**).

The relative content of C18: 2 (n-6) was significantly (*p* < 0.05) decreased (20.56 ± 0.46% to 13.64 ± 2.13%); C18: 1 (n-9) presented an insignificant change. Moreover, the relative contents of C18: 3 (n-6), C20: 4 (n-6), and C22: 6 (n-6) also significantly decreased (*p* < 0.05) from stage A to F (Figure 1A). All referenced UFAs accounted for an even lower relative content at stage F. The oxidation and lipolysis of FA, especially for C18: 2 (n-6) and C18: 3 (n-6), produced alcohols, aldehydes, and ketones, which were responsible for abundant aroma as well as “rancid” off-flavors after storage [15,16]. The change of FA composition was consistent with the increase in TBARS values. Previous studies have presented similar results [16,17].

In our research, sensory factors included taste, tenderness, juiciness, aroma, and appearance (Figure 1B). The previous studies mainly focused on analyzing the sensory change in sauced duck during storage. The change mechanism of sensory quality during marinating, cooking, and drying was revealed in this study. Cooking and drying significantly improved the scores of taste, aroma, and tenderness. The high salt content in the marinating treatment and water losses gave duck meat a firm texture [18]. Sauced duck had a more abundant aroma under the metabolism of microbes and enzymes after a proper storage [16]. However, excessive storage for sauced duck increased the values of T-VBN, TBARS, ammonia, and PUFA metabolites, negatively affecting aroma and taste. The total scores reached a relatively high level after 15 d storage.

### 3.2. The Microbial Biomarkers of Samples Were Identified at Different Stages

**Figure 2:**
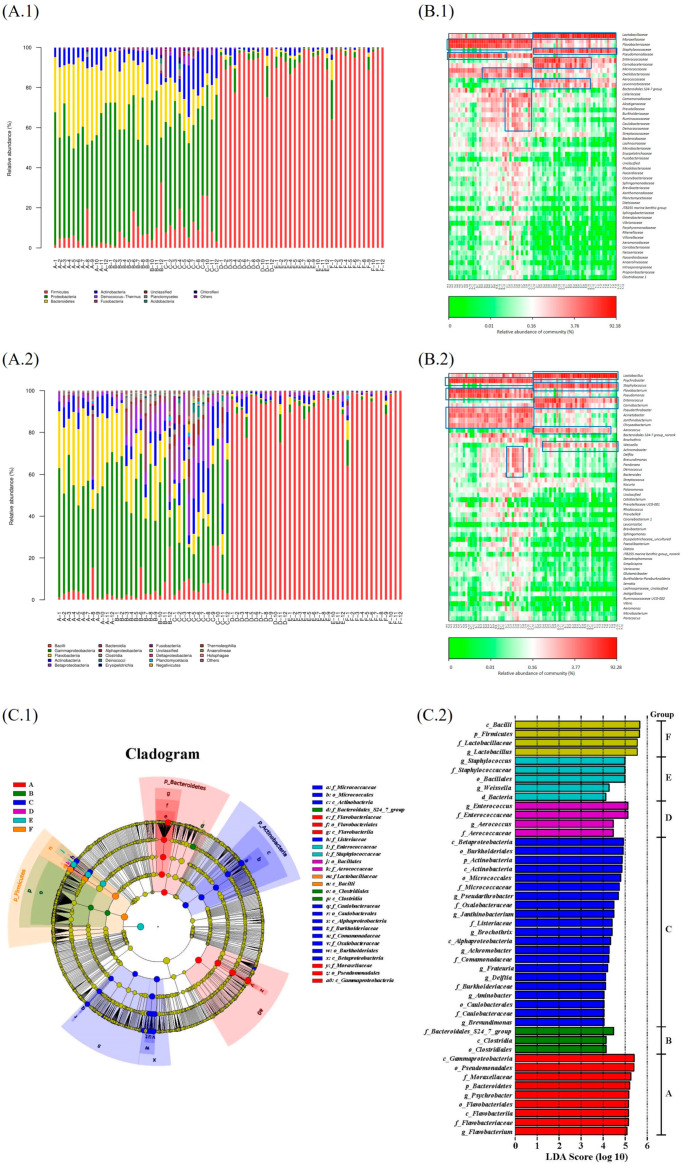
Bacterial flora of samples at different stages; bar plot at family level (**A.1**) and at genus level (**A.2**); heat map at family level (**B.1**) and at genus level (**B.2**); cladogram (**C.1**) and LDA analysis diagram (**C.2**) of bacterial flora at different stages. Note: Different letter (A–F) stands for different stages. Different number (1–12) after letters stands for different repetitions in each stage. The dominant genera and family is marked with blue box.

The rarefaction curves in Figure S1 indicate the reliability of the sequencing results. Figure 2(A.1,A.2) demonstrates the relative proportion of bacteria at different stages. At the family level, *Moraxellaceae*, *Flavobacteriaceae*, and *Pseudomonadaceae* dominated during the stages A–C, which is consistent with [13]. At the genus level, *Psychrobacter*, *Flavobacterium*, and *Pseudomonas* were the dominant genera during stages A–C. In the heat map Figure 2(B.1,B.2), more red spots observed during stages A–C indicate higher bacterial abundance; *Lactobacillus*, *Staphylococcus*, and *Enterococcus* dominated stages D–F. As demonstrated in Figure 2(C.1,C.2), 46 biomarkers were found, and most (n = 21) were detected at stage C. The α-diversity showed the most complex bacterial flora during stages A–C (Figure S2).

Two groups were distinguished in the cluster tree: one comprised mainly samples of stages A–C groups, and the other was almost found during stages D–F. Also, the potential pathogenic biomarkers at stages C (*f_Listeriaceae*), D (*f_Enterococcus* and *g_Enterococcus*), and E (*f_Staphylococcus* and *g_Staphylococcus*) are worthy of note. The sequencing results are consistent with [19]. The duck’s initial microbial load, which is related to poultry health, temporal development, and rearing conditions, may be the essential source of these pathogenic bacteria [9,19]. Also, the variations could be caused by several treatments, such as the slaughtering, slicing, and marinating process or during storage [5]. The optimization of poultry health management and the development of the hygiene level during processing and storage are essential for sauced duck production. However, disinfection and microbial change might eventually influence the flavor development of sauced duck.

### 3.3. The Quantitative and Qualitative Analyses of Flavor Compounds of Samples at Different Stages Were Performed

**Figure 3:**
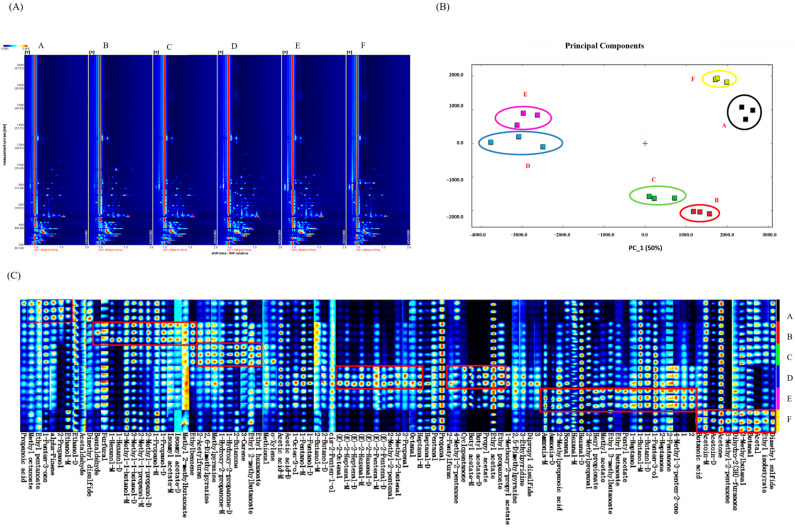
Two-dimensional topographic plot (**A**), fingerprint spectra (**B**), and PCA (**C**) in the GC-IMS results. The flavor markers are marked by red boxes.

Aldehydes, esters, alcohols, and ketones contributed significantly to the sauced duck’s flavor [7]. Aldehydes such as hexanal, octanal, heptanal, and benzaldehyde are the most crucial compounds of sauced duck [20]. In this study, 81 flavor compounds were clarified, including 19 aldehydes, 19 esters, 13 alcohols, 11 ketones, and 4 acids (Figure 3). The flavor description of the main substances (n = 65) is listed in Table 2. Ethanol was the flavor compound with the highest relative contents from stages A to F. The addition of yellow wine brought the meat a mellow aroma and attractive flavor. Hexanal, ethyl acetate, acetone, acetic acid, and propanal were the main flavor compounds, constituting the characteristic flavor of sauced duck. The minimum values of acetone and hexanal as well as the maximum value of ethyl acetate were found at stage E.

According to the odor descriptions in Table 2, such chemical changes gave the samples at stage E a more robust floral and fruity and less pungent aroma, presenting a more desirable flavor. The increase in ammonia was consistent with the T-VBN value. High ammonia contents could exhibit an unpleasant odor [5].

Various flavor markers at different stages of samples were exhibited (Figure 3A). There were plenty of flavor markers at stages A–C and D–F. Ethyl pentanoate, alpha-pinene, and 2-propanol were the flavor markers at stage A. Four flavor markers were screened at both stage B (furfural, 1-hexanol, isoamyl acetate, and methyl 2-methylbutyrate) and stage C (2-acetylfuran, 1-hydroxy-2-propanone, 3-carene, and ethyl hexanoate). Both lipid oxidation and Strecker degradation could produce aldehydes and alcohols, such as 1-octen-3-ol, 1-pentanol, hexanal, and (E)-2-hexenal [16]. The high hexanal content presents a rotten odor in sauced ducks [17,21]. However, the sensory results reflected that the odor of sauced ducks at stage F was acceptable. The interaction of flavor compounds might influence the threshold of off-flavor. The esters, mainly ethyl acetate, were derived from the esterification reactions during the cooking treatment. Interestingly, we found that stage A was closer to stage F in PCA result of flavor compounds (Figure 3B). The dissipation of new flavor markers and enhancement of initial compounds during storage might account for the PCA result [22].

During stages D–F, seven, seven, and four markers were identified. At stage D, butyl acetate, propyl acetate, 1-methoxy-2-propyl acetate, cis-2-penten-1-ol, (E)-2-octenal, 2-propenal, and 2-pentylfuran were found as the flavor markers. Sauced duck samples were rich in carbonyl compounds and amino acid (AA) compounds, which are the primary substrates of the Maillard reaction. Therefore, the contents of furans, including furfural, 2-acetylfuran, 2-pentylfuran, and dihydro-2(3H)-furanone, significantly (*p* < 0.05) increased during stages C–D. At stage E, apparent increases were found in the contents of ammonia, nonanal, hexyl propionate, methyl acetate, pentyl acetate, 1-butanol, and 2-heptanone. At stage F, acetoin, propanal, 3-methyl-2-pentanone, 3-methylbutanal, butanal, and acetal increased. Figure 3C exhibited a decrease in new esters and increase in original acids and alcohols, which might account for such PCA results. Our results differ slightly from previous findings because of different processing materials and methods [4].

### 3.4. The Biomarker 3-Carene Was Clarified Through Correlation Analysis

**Figure 4:**
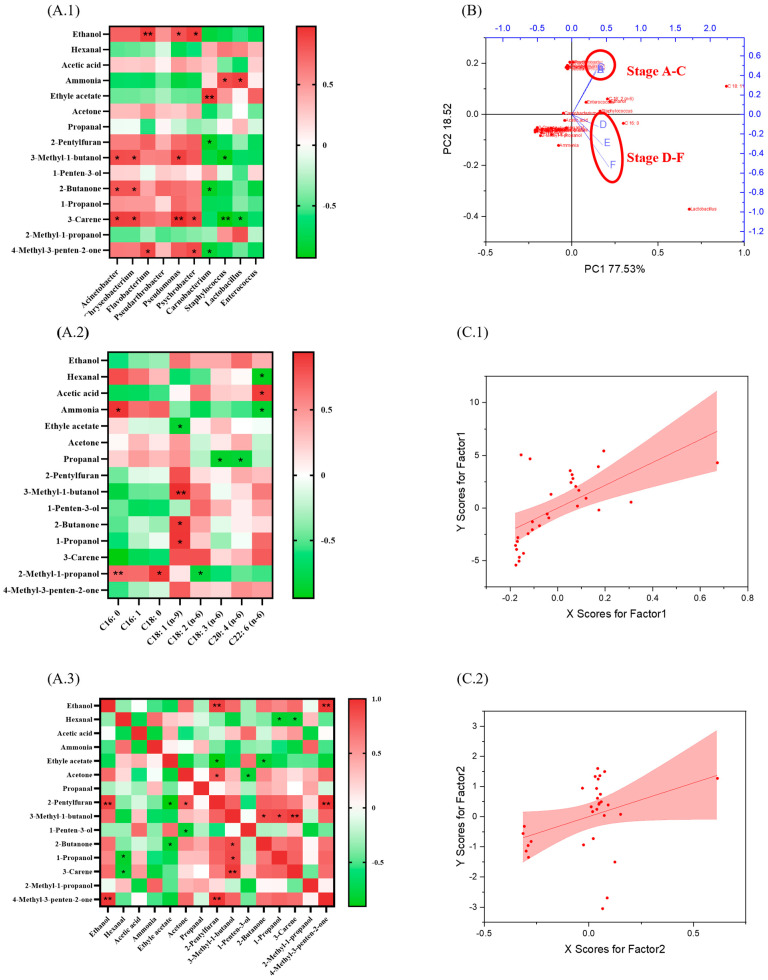
The correlation analysis (**A**), PCA (**B**), and PLS-DA (**C**) results of flavor compounds, dominant bacteria, and free acids. Note: * *p* < 0.05; ** *p* < 0.005. In heat map (**A**), red color stands for positive correlation; green color stands for negative correlation; white color stands for no correlation.

The different thermal processing parameters of marinating, cooking, drying, and storage change accounted for the flavor development of sauced duck. The Pearson correlation analysis results are presented in Figure 4. All dominant bacteria showed significant correlation with the main flavor substances except for *Pseudarthrobacter* and *Enterococcus* (Figure 4(A.1)). The dominant bacteria of stages A–C exhibited mainly positive correlations, which were different from the dominant bacteria of stages D–F. 3-Carene showed significant correlations to six of the ten dominant bacteria. Due to the similar change mechanisms under the processing treatment of samples in this study, 3-carene could be a marker to roughly estimate the change of dominant bacteria. 

In general, terpenes such as 3-carene were mainly derived from spices rather than the flavor metabolites during stages A–F [23]. In this study, the change of C 18: 1 (n-9) presented a high correlation with the main flavor compounds in Figure 4(A.2), a finding that differs from previous studies. As discussed before, the change of C18: 1 (n-9) was insignificant during stages A–F. This different result could be attributed to the stable properties of these flavor compounds as well as the addition of several spices and seasonings. Previous studies reported that C18: 2 (n-6) and C18: 3 (n-6) accounted for several flavor substances because of lipid oxidation and Strecker degradation [7,16,17]. The low relative contents of C18: 2 (n-6) and C18: 3 (n-6) could be an essential reason. The actual numerical relationship and concrete molecular transformation mechanism of main components in Figure 4(A.3) need further verification. Also, a comprehensive analysis of flavor compounds, dominant bacteria, and free acids could draw a similar conclusion (Figure 4B,C). The overall quality change of sauced duck samples during different stages (A–F) exhibited regularity and significant (*p* < 0.05) grouping.

### 3.5. The Potential Chemical Reactions Were Predicted Based on the Previous Analysis

**Figure 5:**
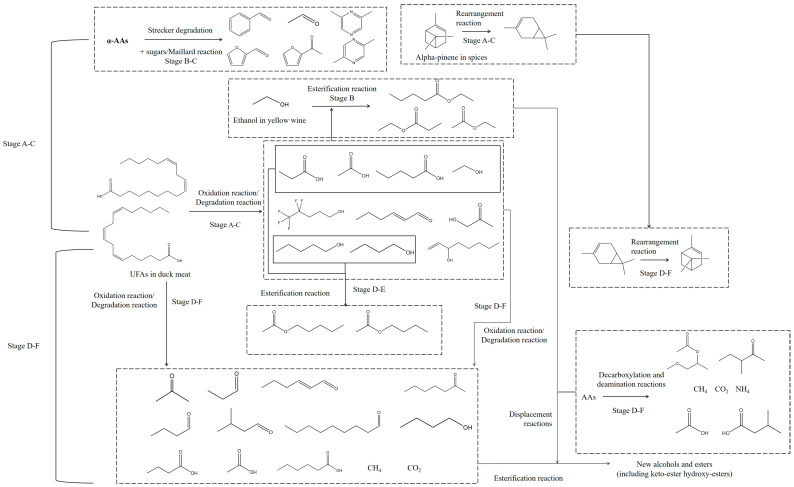
The potential chemical reactions of sauced duck samples during stages A–F [25,26,27].

Overall, our findings are consistent with previous studies [16,24]. The correlations between main flavor compounds (including acids, ketones, alcohols, and other related substances) are presented in Figure 4A. Our prediction was based on the reaction requirement for occurrence and component change during different stages. 2-Pentylfuran and 3-methy-1-butanol were significantly correlated (*p* < 0.05) with more than three compounds. They are regarded as the products of the Maillard reaction and Strecker degradation [7,17]. As shown in Figure 4, 3-methy-1-butanol presented a more significant correlation (*p* < 0.05 or 0.005) with FA and dominant bacteria compared to 2-pentylfuran. Based on the changes in FA and flavor composition results, the potential chemical reactions at different stages are depicted in Figure 5. The complex reactions, such as the Maillard reaction, Strecker degradation, oxidation reaction, and esterification, were the essential reasons for the changes in FA and flavor. As the crucial compounds in flavor development, 2-pentylfuran and 3-methy-1-butanol have a “Butter, Floral” description. Moreover, the content of 3-methy-1-butanol increased significantly from stage A to F, the same as the sensory quality scores. 3-Methy-1-butanol could be an essential indicator in the quality evaluation of sauced ducks.

## 4. Conclusions

The present study investigated the changes in sauced duck across six different stages. The values of edible indexes and FA composition exhibited significant change (*p* < 0.05). The dominant bacteria of stages A–C were *Psychrobacter*, *Flavobacterium*, and *Pseudomonas*. *Lactobacillus*, *Staphylococcus*, and *Enterococcus* dominated during stages D–F. Furan compounds were the primary Maillard reaction products in sauced duck. 3-Methy-1-butanol could serve as an indicator in the quality evaluation of sauced duck.

## Figures and Tables

**Table 1:** Color changes of samples at different stages.

Index	Stage					
	A	B	C	D	E	F
L*	32.1 ± 0.3 ^a^	48.2 ± 0.8 ^f^	46.5 ± 0.3 ^e^	44.3 ± 0.2 ^d^	42.5 ± 0.1 ^c^	41.3 ± 0.1 ^b^
a*	8.2 ± 0.9 ^a^	10.5 ± 0.3 ^b^	11.2 ± 0.6 ^c^	11.5 ± 0.1 ^c^	12.6 ± 0.5 ^d^	12.1 ± 0.3 ^d^
b*	5.8 ± 0.6 ^a^	6.0 ± 1.2 ^b^	7.0 ± 0.4 ^c^	7.8 ± 0.6 ^d^	8.1 ± 0.3 ^d^	8.2 ± 0.3 ^d^
T-VBN (mg/100 g)	5.92 ± 0.42 ^a^	6.85 ± 0.25 ^b^	7.12 ± 0.92 ^b^	20.25 ± 0.89 ^c^	24.58 ± 0.43 ^d^	48.20 ± 1.06 ^e^
TBARS (mg/kg)	0.16 ± 0.00 ^a^	0.27 ± 0.03 ^b^	0.58 ± 0.05 ^c^	0.96 ± 0.08 ^d^	1.37 ± 0.02 ^e^	2.13 ± 0.15 ^f^
TPC (log_10_ CFU/g)	1.82 ± 0.02 ^a^	2.24 ± 0.06 ^b^	2.20 ± 0.05 ^b^	6.85 ± 0.01 ^c^	7.82 ± 0.12 ^d^	8.45 ± 0.08 ^e^

Note: The same letter means the difference is not significant, while a different letter means the difference is significant (*p* < 0.05).

**Table 2:** The main flavor substances in the GC-IMS results.

No.	Compound	CAS	Formula	Description
	Aldehydes (n = 15)			
1	Benzaldehyde	C100527	C_7_H_6_O	Bitter almond, Burnt sugar, Cherry, Malt, Roasted pepper
2	Nonanal	C124196	C_9_H_18_O	Fat, Floral, Green, Lemon
3	(E)-2-Heptenal	C18829555	C_7_H_12_O	Almond, Fat, Fruit
4	Heptanal	C111717	C_7_H_14_O	Citrus, Fat, Green, Nut
5	Hexanal	C66251	C_6_H_12_O	Apple, Fat, Fresh, Green, Oil
6	Pentanal	C110623	C_5_H_10_O	Almond, Bitter, Malt, Oil, Pungent
7	Acetal	C105577	C_6_H_14_O_2_	Creamy, Fruit, Pleasant, Tropical fruit
8	Butanal	C123728	C_4_H_8_O	Banana, Green, Pungent
9	2-Methylpropanal	C78842	C_4_H_8_O	Burnt, Caramel, Cocoa, Green, Malt
10	Propanal	C123386	C_3_H_6_O	Floral, Pungent, Solvent
11	Acetaldehyde	C75070	C_2_H_4_O	Floral, Green apple
12	Methional	C3268493	C_4_H_8_OS	Cooked potato, Soy
13	3-Methyl-2-butenal	C107868	C_5_H_8_O	Almond, Roasted
14	2-Methyl-2-pentenal	C623369	C_6_H_10_O	Fruit
15	Octanal	C124130	C_8_H_16_O	Citrus, Fat, Green, Oil, Pungent
	Esters (n = 17)			
1	Methyl octanoate	C111115	C_9_H_18_O_2_	Fruit, Orange, Wax, Wine
2	(E)-2-Octenal	C2548870	C_8_H_14_O	Dandelion, Fat, Fruit, Grass, Green, Spice
3	Hexyl propionate	C2445763	C_9_H_18_O_2_	Fruit
4	1-Penten-3-ol	C616251	C_5_H_10_O	Butter, Fish, Green, Oxidized, Wet earth
5	Methyl 2-methylbutanoate	C868575	C_6_H_12_O_2_	Apple, Fruit, Green apple, Strawberry
6	Ethyl acetate	C141786	C_4_H_8_O_2_	Aromatic, Brandy, Grape
7	Methyl acetate	C79209	C_3_H_6_O_2_	Ester, Green
8	Ethyl propanoate	C105373	C_5_H_10_O_2_	Apple, Pineapple, Rum, Strawberry
9	Propyl acetate	C109604	C_5_H_10_O_2_	Celery, Floral, Pear, Red fruit
10	Ethyl 3-methylbutanoate	C108645	C_7_H_14_O_2_	Apple, Fruit, Pineapple, Sour
11	Ethyl 2-methylbutanoate	C7452791	C_7_H_14_O_2_	Apple, Ester, Green apple, Kiwi, Strawberry
12	Isoamyl acetate	C123922	C_7_H_14_O_2_	Apple, Banana, Pear
13	Ethyl butanoate	C105544	C_6_H_12_O_2_	Apple, Butter, Cheese, Pineapple, Strawberry
14	Butyl acetate	C123864	C_6_H_12_O_2_	Apple, Banana
15	Pentyl acetate	C628637	C_7_H_14_O_2_	Apple, Banana, Pear
16	Ethyl hexanoate	C123660	C_8_H_16_O_2_	Apple peel, Brandy, Fruit gum, Overripe fruit, Pineapple
17	Ethyl pentanoate	C539822	C_7_H_14_O_2_	Apple, Dry fish, Herb, Nut, Yeast
	Alcohols (n = 9)			
1	1-Octen-3-ol	C3391864	C_8_H_16_O	Cucumber, Earth, Fat, Floral, Mushroom
2	1-Hexanol	C111273	C_6_H_14_O	Banana, Flower, Grass, Herb
3	Acetoin	C513860	C_4_H_8_O_2_	Butter, Creamy, Green pepper
4	1-Pentanol	C71410	C_5_H_12_O	Balsamic, Fruit, Green, Pungent, Yeast
5	3-Methyl-1-butanol	C123513	C_5_H_12_O	Burnt, Cocoa, Floral, Malt
6	1-Butanol	C71363	C_4_H_10_O	Fruit
7	2-Methyl-1-propanol	C78831	C_4_H_10_O	Apple, Bitter, Cocoa, Wine
8	1-Propanol	C71238	C_3_H_8_O	Alcohol, Candy, Pungent
9	2-Propanol	C67630	C_3_H_8_O	Floral
	Ketones (n = 8)			
1	1-Hydroxy-2-propanone	C116096	C_3_H_6_O_2_	Butter, Herb, Malt, Pungent
2	2-Heptanone	C110430	C_7_H_14_O	Blue cheese, Fruit, Green, Nut, Spice
3	1-Penten-3-one	C1629589	C_5_H_8_O	Fish, Green, Mustard, Pungent
4	2-Pentanone	C107879	C_5_H_10_O	Fruit, Pungent
5	2-Butanone	C78933	C_4_H_8_O	Fragrant, Fruit, Pleasant
6	Acetone	C67641	C_3_H_6_O	Pungent
7	Cyclopentanone	C120923	C_5_H_8_O	Mint, Cool
8	Dihydro-2(3H)-furanone	C96480	C_4_H_6_O_2_	Caramel, Cheese, Roasted nut
	Acids (n = 4)			
1	Propanoic acid	C79094	C_3_H_6_O_2_	Fat, Fruit, Pungent, Silage, Soy
2	Butanoic acid	C107926	C_4_H_8_O_2_	Butter, Cheese, Sour
3	Acetic acid	C64197	C_2_H_4_O_2_	Acid, Fruit, Pungent, Sour, Vinegar
4	2-Methylpropanoic acid	C79312	C_4_H_8_O_2_	Burnt, Butter, Cheese, Sweat
	Others (n = 12)			
1	2,6-Dimethylpyrazine	C108509	C_6_H_8_N_2_	Cocoa, Coffee, Green, Roast beef, Roasted nut
2	Methylpyrazine	C109080	C_5_H_6_N_2_	Cocoa, Green, Hazelnut, Popcorn, Roasted
3	2-Pentylfuran	C3777693	C_9_H_14_O	Butter, Floral, Fruit, Green bean
4	3-Carene	C13466789	C_10_H_16_	Lemon
5	Dimethyl disulfide	C624920	C_2_H_6_S_2_	Cabbage, Garlic, Onion
6	alpha-Pinene	C80568	C_10_H_16_	Cedarwood, Pine, Sharp
7	Dimethyl sulfide	C75183	C_2_H_6_S	Cabbage, Organic, Sulfur, Wet earth
8	Furfural	C98011	C_5_H_4_O_2_	Almond, Baked potatoes, Bread, Burnt, Spice
9	Dipropyl disulfide	C629196	C_6_H_14_S_2_	Cooked meat, Garlic, Onion, Pungent, Sulfur
10	3-Ethylpyridine	C536787	C_7_H_9_N	Nuts
11	2,5-Dimethylpyrazine	C123320	C_6_H_8_N_2_	Cocoa, Roast neef, Roasted nut
12	2-Acetylfuran	C1192627	C_6_H_6_O_2_	Balsamic, Cocoa, Coffee

## Data Availability

The original contributions presented in the study are included in the article/Supplementary Materials, further inquiries can be directed to the corresponding authors.

## Supplementary Materials

The following supporting information can be downloaded at https://www.mdpi.com/article/10.3390/foods14050834/s1. Figure S1: The α-rarefaction curve for different stages of bacterial flora; Figure S2: Diversity analysis of bacterial flora; Figure S3: Qualitative analysis of flavor substances; Table S1: Criteria for the sauced duck samples’ sensory evaluation; Table S2: Main compounds responsible for sensory attribute.

## Abbreviations

The following abbreviations are used in this manuscript:

AA	amino acid
ANOSIM	analysis of similarities
CTAB/SDS	cetyltrimethylammonium bromide/sodium docecyl sulfate-based
FA	free fatty acid
GC-IMS	gas chromatography–ion mobility spectrometry
GC-MS	gas chromatography–mass spectrometry
HCl	hydrochloric acid
LDA	linear discriminant analysis
MDA	malondialdehyde
MgO	magnesium oxide
MRPP	multi-response permutation procedure
MUFA	mono-unsaturated fatty acid
NaCl	sodium chloride
PCA	principal component analysis
PCoA	principal coordinate analysis
PLS-DA	partial least squares–discriminant analysis
PUFA	poly-unsaturated fatty acid
SFA	saturated fatty acid
Stage A	samples collected after marinating and before cooking
Stage B	samples collected after cooking and before drying
Stage C	samples collected after drying and before storage
Stage D	samples stored after 5 d
Stage E	samples stored after 10 d
Stage F	samples stored after 15 d
TBARS	thiobarbituric acid reactive substance
TPC	total plate count
T-VBN	total volatile basic nitrogen
UPGMA	unweighted pair group method with arithmetic mean
UFA	unsaturated fatty acid
