# Supplementary material for: The Overall Quality Changes of Chinese Sauced Ducks at Different Stages During Processing and Storage
Source: Foods. 2025 Feb 28;14(5):834. doi: 10.3390/foods14050834 (PMC11899615; doi:10.3390/foods14050834)
Supplement: Supplementary file 1 [file foods-14-00834-s001.zip › foods-3492662-supplementary.pdf]

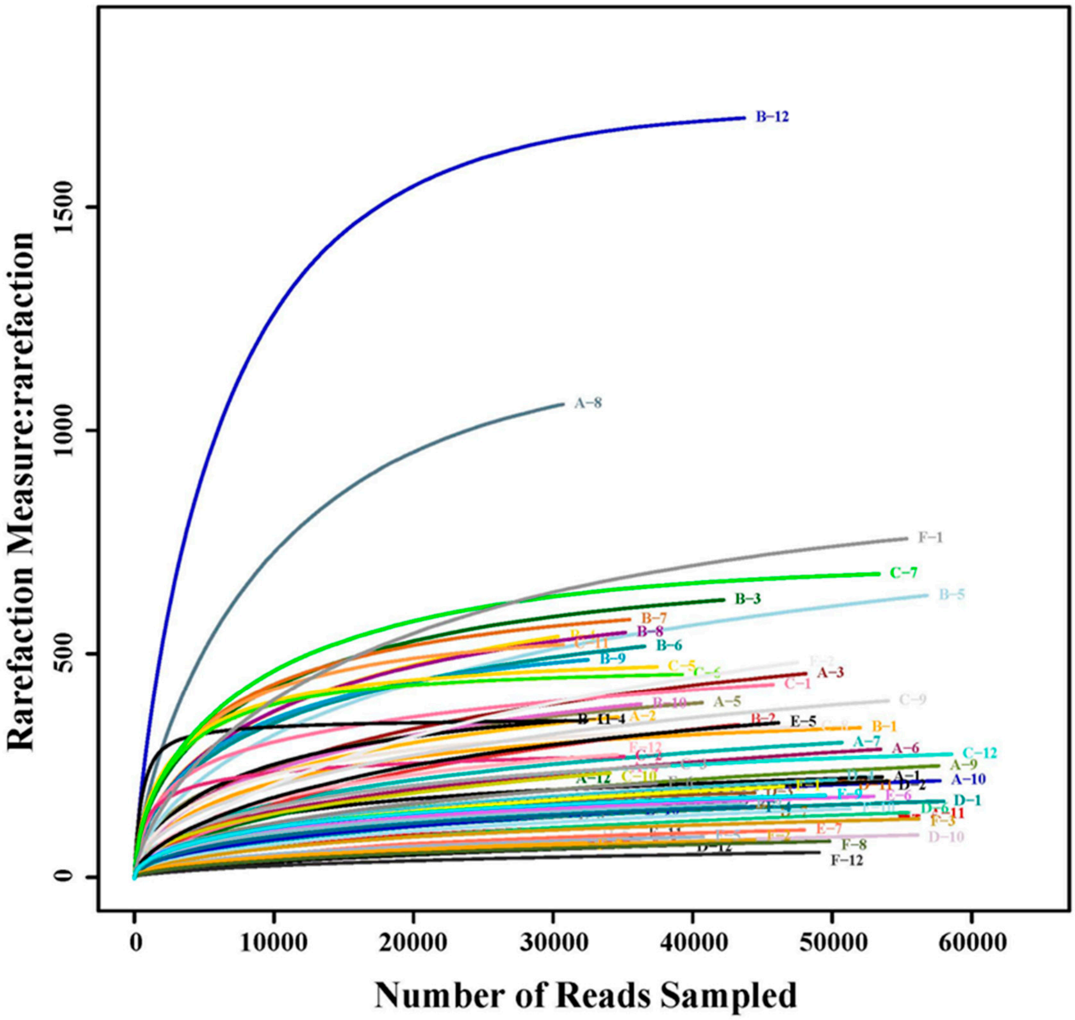

Figure. S1 The α-rarefaction curve for different stages of bacterial flora.

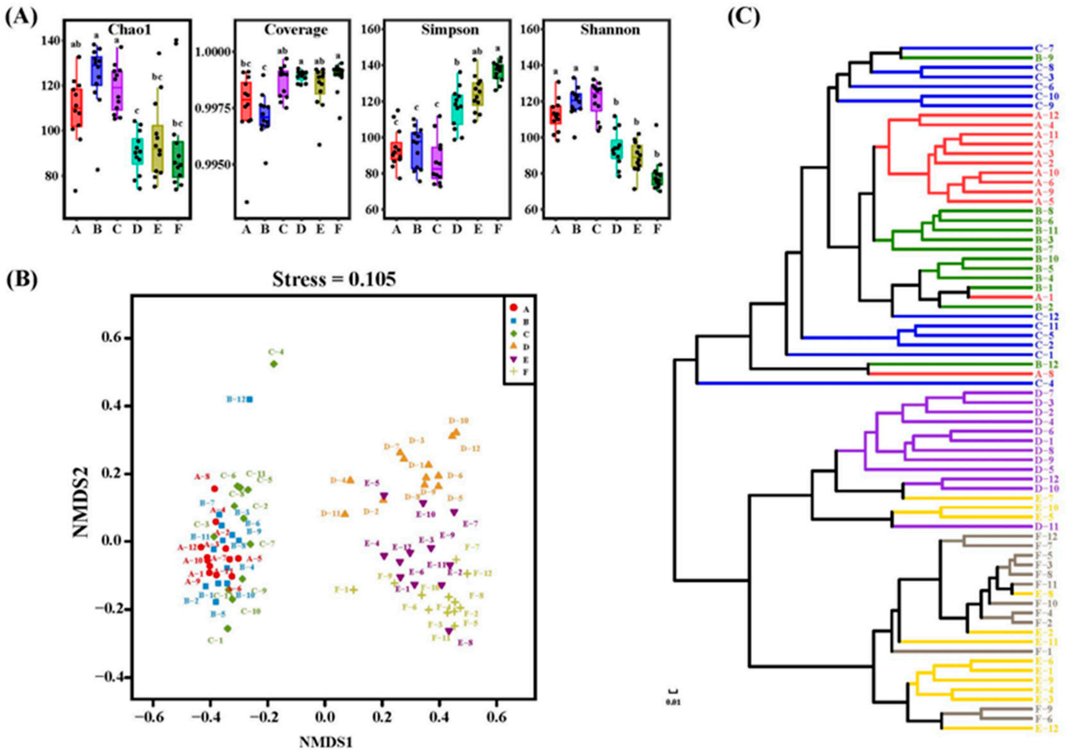

Figure. S2 Diversity analysis of bacterial flora at different stages.（A): α-Diversity analysis （B): NMDS analysis （C): Cluster tree

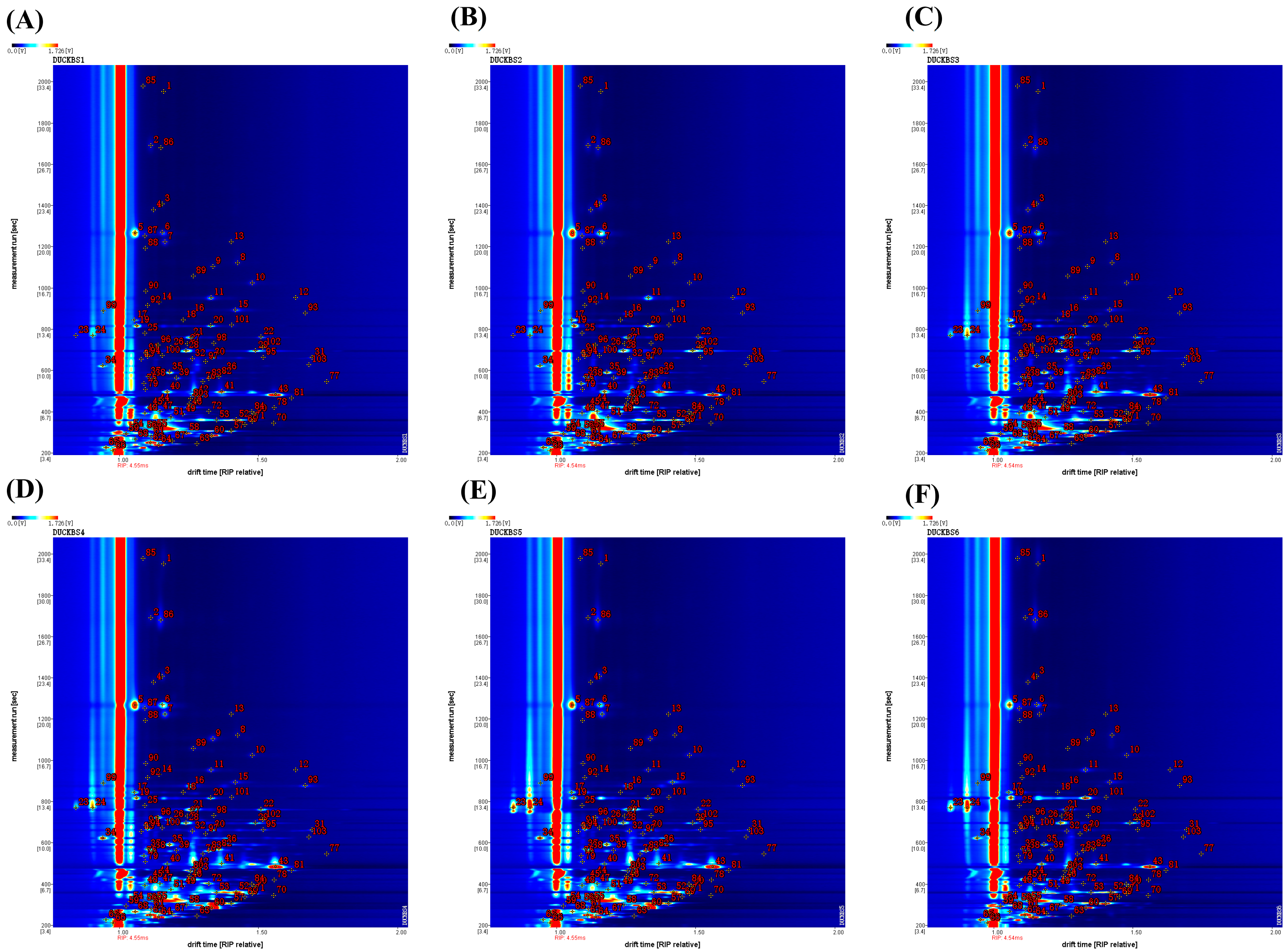

Figure. S3 Qualitative analysis of flavor substances in samples at different stages A-F

Table S1 Criteria for the sauced duck samples' sensory evaluation

Parameter	Grading criteria	Grade (1-9)
Taste	Overall liking	1 extremely dislike, 3 dislike, 5 neither like nor dislike, 7 like, 9 extremely like
Tenderness	Overall texture of meat	1 extremely tough, 3 tough, 5 neither tough nor tender, 7 tender， 9 extremely tender
Juiciness	The moisture released	1 extremely low, 3 low, 5 neither low nor high, 7 high, 9 extremely high
Aroma	Attractive or unpleasant smell	1 extremely dislike, 3 dislike, 5 neither like nor dislike, 7 like, 9 extremely like
Appearance	Color and shape	1 extremely undesirable, 3 undesirable, 5 neither desirable nor undesirable, 7 desirable, 9 extremely desirable

Table S2 Main compounds responsible for sensory attribute

No.	Attribute	Compounds
1	Fruit (n = 18)	Hexanal, Nonanal, (E)-2-Heptenal, 2-Methyl-2-pentenal, Hexyl propionate, 2-Methyl-1-propanol, Acetal, Butanal, 2-Pentanone, Propanoic acid, Acetic acid, 2-Pentylfuran, Isoamyl acetate, Ethyl butanoate, Butyl acetate, Pentyl acetate, Methyl octanoate, Propyl acetate
2	Floral (n = 8)	Propanal, Acetaldehyde, Nonanal, Propyl acetate, 1-Octen-3-ol, 3-Methyl-1-butanol, 2-Propanol,
3	Fat (n = 8)	Hexanal， Nonanal， (E)-2-Heptenal, Heptanal, Octanal, (E)-2-Octenal, Propanoic acid，1-Octen-3-ol，Propanoic acid, 2-Pentylfuran
4	Nut (n = 8)	Heptanal, Ethyl pentanoate, 2-Heptanone, Dihy-dro-2(3H)-furanone, 2,6-Dimethylpyrazine, Methylpyrazine, 3-Ethylpyridine, 2,5-Dimethylpyrazine
5	Cocoa (n = 7)	2-Methylpropanal, 3-Methyl-1-butanol, 2-Methyl-1-propanol, 2,6-Dimethylpyrazine, Methylpyrazine, 2,5-Dimethylpyrazine, 2-Acetylfuran
6	Butter (n = 7)	1-Penten-3-ol, Ethyl butanoate, Acetoin, 1-Hydroxy-2-propanone, Butanoic acid, 2-Methylpropanoic acid, 2-Pentylfuran
